# Asymmetry in kinematics of dominant/nondominant lower limbs in central and lateral positioned college and sub-elite soccer players

**DOI:** 10.1371/journal.pone.0304511

**Published:** 2024-06-07

**Authors:** Francisco Beron-Vera, Sergio A. Lemus, Ahmed O. Mahmoud, Pedro Beron-Vera, Alexander Ezzy, Cheng-Bang Chen, Bryan J. Mann, Francesco Travascio

**Affiliations:** 1 Department of Mechanical and Aerospace Engineering, University of Miami, Coral Gables, FL, United States of America; 2 Department of Physics, University of Miami, Coral Gables, FL, United States of America; 3 Department of Industrial Engineering, University of Miami, Coral Gables, FL, United States of America; 4 Department of Kinesiology and Sport Management, Texas A&M University, College Station, TX, United States of America; 5 Department of Orthopaedics, University of Miami, Miami, FL, United States of America; 6 Max Biedermann Institute for Biomechanics at Mount Sinai Medical Center, Miami Beach, FL, United States of America; Ningbo University, CHINA

## Abstract

Change of direction, stops, and pivots are among the most common non-contact movements associated with anterior cruciate ligament (ACL) injuries in soccer. By observing these dynamic movements, clinicians recognize abnormal kinematic patterns that contribute to ACL tears such as increased knee valgus or reduced knee flexion. Different motions and physical demands are observed across playing positions, which may result in varied lower limb kinematic patterns. In the present study, 28 college and sub-elite soccer players performed four dynamic motions (change of direction with and without ball, header, and instep kick) with the goal of examining the effect of on-field positioning, leg dominance, and gender in lower body kinematics. Motion capture software monitored joint angles in the knee, hip, and ankle. A three-way ANOVA showed significant differences in each category. Remarkably, centrally positioned players displayed significantly greater knee adduction (5° difference, p = 0.013), hip flexion (9° difference, p = 0.034), hip adduction (7° difference, p = 0.016), and dorsiflexion (12° difference, p = 0.022) when performing the instep kick in comparison to their laterally positioned counterparts. These findings suggest that central players tend to exhibit a greater range of motion when performing an instep kicking task compared to laterally positioned players. At a competitive level, this discrepancy could potentially lead to differences in lower limb muscle development among on-field positions. Accordingly, it is suggested to implement position-specific prevention programs to address these asymmetries in lower limb kinematics, which can help mitigate dangerous kinematic patterns and consequently reduce the risk of ACL injury in soccer players.

## 1. Introduction

Soccer is one of the most popular sports in the world. There are over 200 million active soccer players worldwide [1] and 1.5 billion people (approximately 19% of the world’s population) watched the 2022 FIFA World Cup Final [2]. Due to its dynamic nature, soccer demands high levels of physical, technical, and tactical skills. Consequently, soccer has a high rate of injury that can go up to 45.2 injuries per 1000 hours of playing time [3]. Over two-thirds of these injuries happen in the lower body, specifically in knees and ankles [3]. These lower body injuries stem from the unique demands of soccer-specific movements, including kicking, sudden changes in direction, and player-to-player contact [4, 5]. One of the most common lower body injuries is the ACL tear, which accounts for 1.3% and 3.7% of all injuries in collegiate men’s and women’s soccer, respectively, and 2.6% of all sport related injuries in the U.S [6]. ACL tears have significant consequences for athletes, including prolonged periods of inactivity during recovery, potential injury reoccurrence after clearance, and, in severe cases, retirement from competitive-level sports [7]. Current preventative measures for ACL injuries in sports include neuromuscular training programs [8, 9], proper technique instruction [10, 11], strength and conditioning [12], and biomechanical analysis and feedback [13, 14].

To better understand the biomechanical factors contributing to ACL tears in soccer, researchers have focused their attention on the dynamic movements that preceded the injury. Previous studies have evaluated whether there was contact at the instant the injury occurred, as well as the action being performed by the affected athlete. Boden et.al analyzed 100 injuries for ACL injury cause, with a non-contact mechanism reported in 72% of the cases [15]. Another study investigated 105 soccer ACL injuries, finding that 83.8% were non-contact [16]. As shown in the literature, most ACL injuries are from non-contact movements and occur during sudden changes in direction, pivoting, and deceleration [17]. These dynamic motions are highly prevalent in soccer; a video analysis study in 2020 reviewed 148 ACL injuries across 10 seasons of professional Italian soccer. The analysis by Villas et.al found that the most common non-contact movements that led to ACL injuries were changing direction while sprinting, change of direction when dribbling the ball, landing after performing a header, and regaining balance after a shot [18]. Similarly, Faunø et.al found that 63% of soccer ACL injuries involved the player intending to change direction while running, and 13% when landing from a header without having been pushed or disturbed in the air prior to landing [16].

By observing the most prevalent dynamic movements that precede ACL injuries in soccer, clinicians have identified abnormal patterns such as increased knee valgus or reduced knee flexion during a landing maneuver, or excessive internal rotation during cutting [19–21]. Monitoring lower body kinematics helps to quantify and compare these patterns. Gender has been established as a factor for an increased ACL injury risk. Female athletes participating in cutting and jumping sports have been reported to have a 4 to 6 times greater chance of tearing their ACL than males [22, 23]. This is attributed to a combination of anatomical and biomechanical characteristics that cause increased knee valgus and hip internal rotation [13, 24]. Another ACL injury risk factor studied in soccer is leg dominance. Soccer players generally have a preferred side to kick the ball, which leads to specific mechanics of injury [25]. Leg dominance asymmetry contributes to the imbalance of weight distribution between both sides of the body, leading to muscular imbalance and consequently increasing risk of lower body injuries [26–29]. The occurrence of ACL injuries in soccer shows to be more common on the dominant side for males compared to 32% of females [30, 31]. Furthermore, the on-field position can potentially be a factor that influences the risk of ACL injuries in soccer. Different positions require different movement patterns and physical demands during a soccer game. Generally, central positioned players (strikers, center forward, midfielders, center backs, sweeper) have more turning and cutting situations during a game compared to a lateral positioned player (winger, fullbacks, wide midfielders, wing back). While these differences between central and lateral soccer players have been covered in the literature [32–36], the on-field position has not been studied as a factor of ACL injury risk, and consequently ACL injury programs have not been focused on the specific task of the player on the field.

Accordingly, the present study focused on the effects of on-field position and dominant leg asymmetry on lower body joint angles of college and sub-elite soccer players. The aim was to detect relationships that can be used as an indicator for potential injury. Consequently, practitioners can design ACL prevention programs based on the soccer player’s position. To the authors’ best knowledge, this is the first known study that investigates lower limb kinematics and specifically accounts for potential differences across leg dominance and on-field position. We hypothesized that on-field position and leg dominance will have a significant effect in hip, knee, and ankle joint angles. Specifically, we expected centrally positioned players to have a greater overall range of motion.

## 2. Materials & methods

### 2.1 Subjects

The procedures and methods used in this study were approved by the Internal Review Board, with approval number #20230127. All participants were informed of the study methods and provided written consent prior to beginning the experiment. A total of 28 (n = 28) participants aged between 19 and 36 years (23 ± 6.1 years) were recruited from two soccer teams. Female participants (n = 14) were recruited from a National Collegiate Athletic Association (NCAA) Division I soccer team. Reported average weight and height for women was 63.3 ± 4.71 kg and 1.69 ± 0.08 m, respectively. Male participants (n = 14) were recruited from a semi-professional soccer team participating in the United Premier Soccer League (UPSL). Men reported an average weight and height of 75.9 ± 8.74 kg and 1.80 ± 0.08 m, respectively. Enrollment began on April 4, 2023, and ended on May 30, 2023. Table 1 presents mean and standard deviation values for weight and height, further categorizing the participants by gender and on-field positioning. Table 2 presents the race and ethnicity reported as a percentage based on the total of participants. Inclusion criteria for both male and female participants consisted of adults aged 18 or older, with no history of musculoskeletal or any medical conditions potentially affecting standard soccer kinematics; former or current participation in either college or professional soccer; a minimum of three soccer practices per week led by a professional coach; and at least one competitive league game per week at the time of data collection. The level of the participants differs from an elite or professional level since none of the players belonged to clubs participating in US professional leagues [37]. Instead, the present study recruited participants from the NCAA division 1 and UPSL, considered college and semi-professional leagues respectively [38].

**Table 1:** Participant demographic table. Average age, weight, and height of the participants, further categorized by their gender and on-field positioning.

		Age [y.o.]	Weight [kg]	Height [m]
**Men (n = 14)**	**Lateral (n = 5)**	28.8 ± 3.9	72.6 ± 7.4	1.77 ± 0.02
	**Central (n = 9)**	26.7 ± 3.6	77.8 ± 9.3	1.81 ± 0.09
	**Subtotal**	27.8 ± 3.8	75.2 ± 8.4	1.79 ± 0.06
**Women (n = 14)**	**Lateral (n = 6)**	20.3 ± 0.8	65.8 ± 7.3	1.58 ± 0.03
	**Central (n = 8)**	19.9 ± 0.6	62.9 ± 5.1	1.70 ± 0.07
	**Subtotal**	20.1 ± 0.7	64.4 ± 6.2	1.64 ± 0.05

**Table 2:** Summary of subjects’ race and ethnicity. All the data are reported as number of subjects and percentages based on the total (n = 28).

	Hispanic or Latino	White alone, not Hispanic or Latino	Black or African American	Asian	Two or more races
**Male (n = 14)**	6 (42.9%)	6 (42.9%)	1 (7.1%)	0 (0.0%)	1 (7.1%)
**Female (n = 14)**	2 (14.3%)	7 (50.0%)	3 (21.4%)	2 (14.3%)	0 (0.0%)
**Subtotal**	8 (28.6%)	13 (46.4%)	4 (14.3%)	2 (7.1%)	1 (3.6%)

### 2.2 In-game movement replication

**Fig 1:**
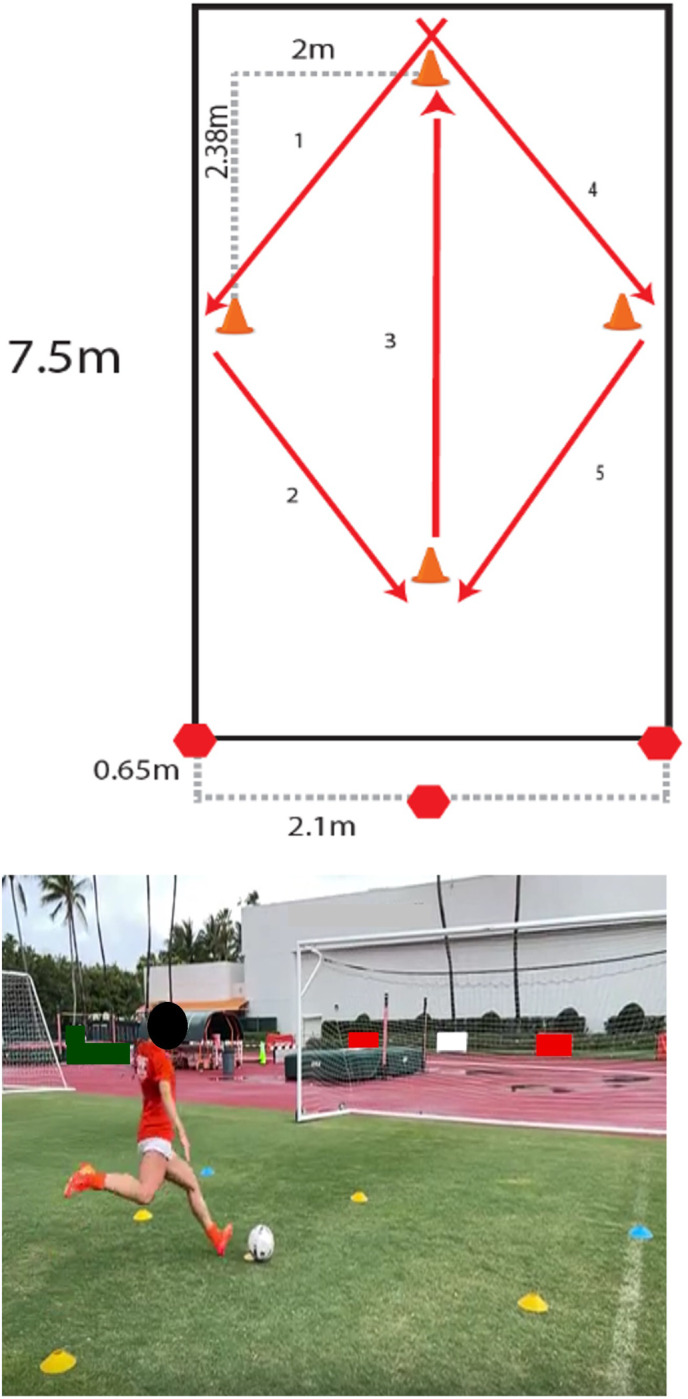
Motion paths and perimeter setup. Dimensions of the perimeter used to recreate motions, along with an image of a participant carrying out an instep kick with this perimeter setup.

Participants were instructed to perform in-game soccer movements associated with non-contact ACL injuries based on the research of Villas et. al and Faunø et.al [16, 18]: changing direction while sprinting, change of direction when dribbling the ball, landing after performing a header, and regaining balance after an instep kick. These four in-game movements were replicated by participants in the present study. Each in-game movement (change of direction, dribble, header, instep kick) was carried out three times by every participant. For change of direction and dribbling, movements were performed three times per direction: cutting from left to right and vice versa. All in-game movements took place within a set perimeter (see Fig 1) to facilitate kinematic data collection. Study coordinators instructed participants to perform the movements as designed for each motion and within the measured perimeter to ensure the consistency in terms of the players’ displacement. The red arrows in Fig 1 represent the path that players were asked to take depending on the executed movement; the diagonal ones were used for change of direction and dribble (arrows 1, 2, 4, and 5). All paths were the same length for each respective movement recorded. For the header and instep kick, the participants went along the path described by arrow 3 and executed the action at the center of the perimeter. These paths were all marked with cones to guide the participant to the appropriate path. The red hexagons at the bottom of the Figure represent the camera setup to record kinematic data.

### 2.3 Joint angle measurement

**Fig 2:**
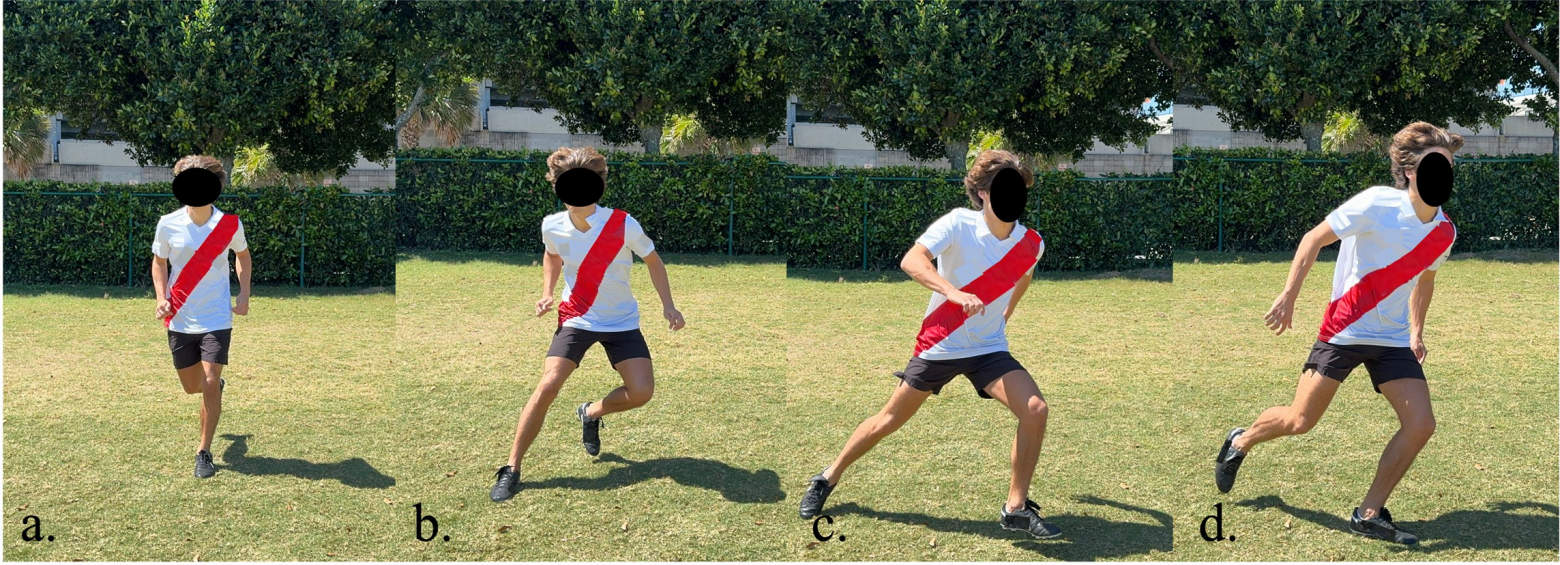
Change of direction & dribble phases. Frames that make up the phases of both the change of direction and dribble tasks, the second phase was analyzed (b-c).

**Fig 3:**
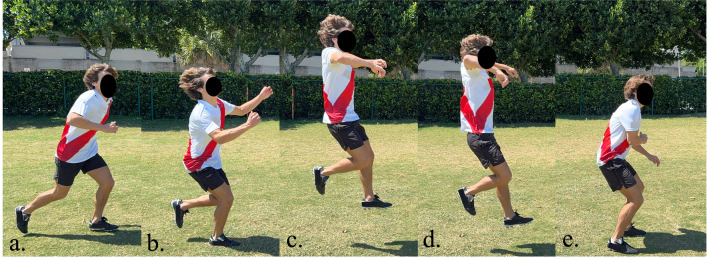
Header phases. Frames that make up the phases of the header task, the third phase was analyzed (d-e).

**Fig 4:**
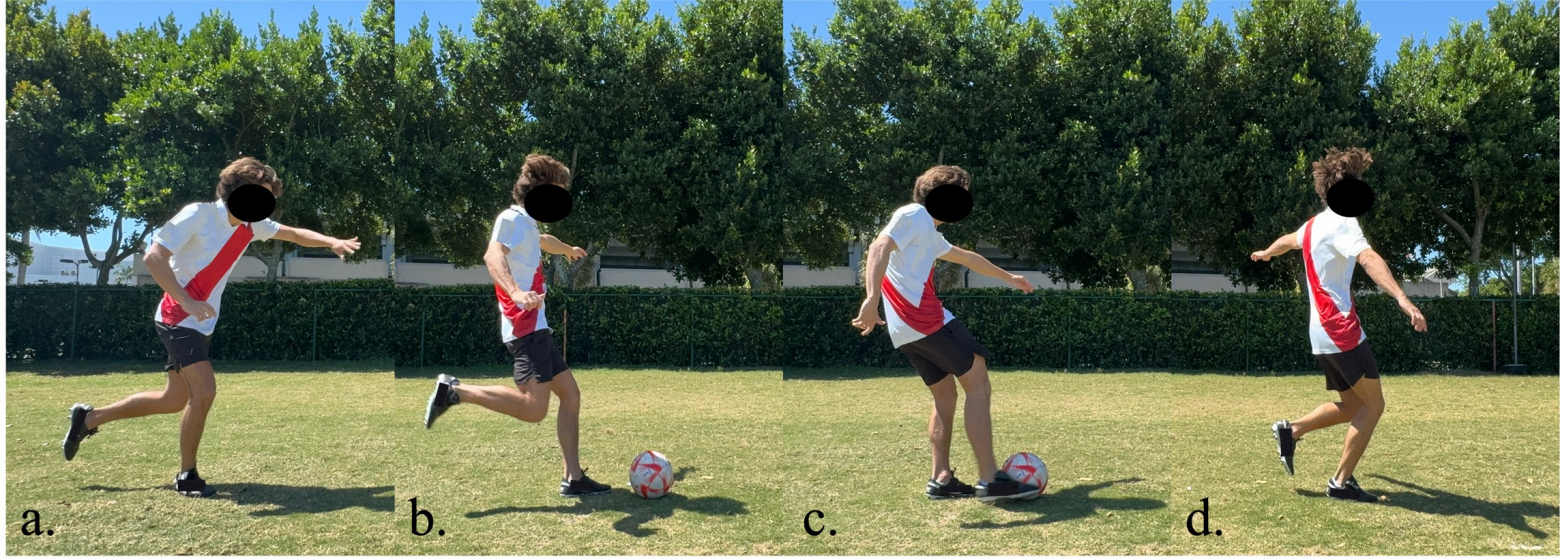
Instep kick phases. Frames that make up the phases of the instep kick task, the second phase was analyzed (b-c).

Kinematic measurements were monitored via OpenCap (1.6, Model Health Inc, California, USA), a marker-less motion capture platform that computes body kinematics by processing videos recorded from synchronized IOS devices using neural networks and musculoskeletal simulation. [39–42]. Two iPhones 13 Max © and an iPad Pro 3^rd^ generation © (Apple Inc., California, USA) were mounted on tripods at a natural grass soccer field, facing a center point, as illustrated in Fig 1. The perimeter was defined to optimize the camera’s position and reduce the likelihood of participants leaving the range of focus. Subject calibration and motions were performed consecutively by the participant, with rest supplied ad libitum between recordings. All motions were broken into phases depicted in Figs 2–4 by selecting 4 relevant frames from the motion simulations.

The change of direction and dribble motions are identical, asides from the inclusion/exclusion of the ball, thus the same phases were selected for them and are described in Fig 2. The first phase starts when the last step leading to the foot plant, to execute direction change, commences upward (Fig 2A) until the instant this planting occurs (Fig 2B). The second phase, or propulsive phase, occurs within the range of motion (Fig 2B and 2C) which is from the instant the foot is planted until it is released to commence the change of direction. The final phase is simply the completion of this step exiting the direction change foot plant (Fig 2C and 2D).

Similar phase selection was performed for the remaining motions, the header task phase breakdown is shown in Fig 3. The first phase was taken from the last step (Fig 3A) leading to when the participants’ feet were planted to commence the jump (Fig 3B), while the second phase was from this plant up until the instant prior to the landing (Fig 3D). The last phase was the landing phase, from the instant prior to the landing until the landing (Fig 3D and 3E).

Likewise, the frames for the phases in the instep kick maneuver are depicted in Fig 4. The instep kick phases breakdown commences with the final step prior to the nondominant foot plant for the shot up (Fig 4A) until the moment this foot is planted (Fig 4B). The following phase was defined from the foot plant up until the moment the participant impacts the ball (Fig 4B and 4C,) this was followed by the third and final phase which was the landing phase (Fig 4C and 4D).

The peak angle values were collected only for relevant phases, such as: propulsive phase for dribble and change of direction (Fig 2B and 2C), landing phase for header (Fig 3D and 3E) and supporting foot planting for shot (Fig 4B and 4C). Kinematics were measured only for the supporting foot during the change of direction, dribble, and instep kick. For the header, both legs were measured at the moment of landing. The recorded motions were then loaded onto OpenSim [43]. Particularly, a musculoskeletal model developed by Lai et. al [44, 45] was integrated to acquire lower limb joint angles. The present study investigated a total of 7 joint angles; 4 for the hip: sagittal flexion/extension, adduction, abduction, and transverse internal rotation; 2 for ankle: dorsiflexion, and transverse subtalar pronation; as well as 2 for knee: sagittal flexion/extension, adduction (valgus), and abduction (varus). All angles were measured for both the participants’ dominant and nondominant sides.

### 2.4 Statistical analysis

Statistical analysis was performed through Minitab (21.1.1, Minitab LLC, Pennsylvania, USA). For all the statistical analyses performed, the level of significance was set at 95% (α = 0.05). All motions were post-processed at their phase of interest via MATLAB© R2023a (MathWorks Inc. ©, Massachusetts, USA). A minimum and a maximum value were obtained, as well as overall mean values across all participants per motion’s phase of interest and joint angle. For comparisons between joint angles of specific movements, a three-way analysis of variance (ANOVA) was conducted for header, change of direction, and dribble motions. For the in-step kick, a two-way ANOVA was conducted since only the shot completed with the dominant foot was measured. To address the issue of missing values, which arose due to absent angle readings (26 missing values out of 3024 records), a general linear model (GLM) was adopted for the ANOVA. To ensure the accuracy of our analysis, Grubb’s tests were performed to remove outliers within our dataset. The factors included in the ANOVA were gender (14 men and 14 women), reported position on the field as a soccer player (11 lateral and 17 central), and dominant leg side of the body (dominant vs. non-dominant, which included 23 right side and 5 left side). To meet the assumptions of the ANOVA, the homogeneity of variances (residuals) within each group was assessed using the Levine test, and the normal distribution of residuals was evaluated using the Shapiro-Wilk test. After conducting the ANOVA, post hoc tests, including Tukey’s and Fisher’s tests, were conducted for pairwise comparisons to identify which groups were significantly different. Finally, a post-hoc power analysis was conducted using G*Power (version 3.1) [46] to assess the statistical power based on the sample size for each joint angle considering gender, leg dominance, and on-field position as the main effects.

## 3. Results

Results were categorized based on the four replicated in-game soccer motions previously detailed: change of direction, dribble, header, and instep kick. P-values, Tukey and Fisher groupings, and significant factors (p < 0.05) are detailed in Tables 3–6. The post-hoc power analysis indicated a power equal to or greater than 95% for inferring statistical differences in each significant peak joint angle, based on gender, leg dominance, and on-field position.

**Table 3:** Change of direction statistics result summary. ’C’ denotes central, ’L’ lateral, ’F’ female, ’M’ male, ’D’ dominant, and ’ND’ nondominant.

Change of Direction	Hip abduction				Internal hip rotation		Subtalar pronation		Knee abduction			
**ANOVA p-value**	0.017				0.002		0.002		< 0.001		0.017	
**Factor**	Lateral-Central * Gender				Gender		Gender		Gender		Leg	
	C-F	C-M	L-F	L-M	F	M	F	M	F	M	D	ND
**Tukey Grouping**	A	A	A	A	A	B	B	A	B	A	B	A
**Fisher Grouping**	A	B	A B	A B	A	B	B	A	B	A	B	A
**Mean (°)**	-29.71	-36.06	-32.62	-32.25	13.92	6.39	-17.95	-8.97	6.32	10.01	7.31	9.02

**Table 4:** Dribble statistics results summary. ’C’ denotes central, ’L’ lateral, ’F’ female, ’M’ male, ’D’ dominant, and ’ND’ nondominant.

Dribble	Hip abduction		Internal hip rotation		Dorsiflexion						Subtalar pronation		Knee abduction	
**ANOVA p-value**	0.032		<0.001		0.035		0.033				0.013		<0.001	
**Factor**	Leg		Gender		Leg		Lateral-Central* Gender				Gender		Gender	
	D	ND	F	M	D	ND	C-F	C-M	L-F	L-M	F	M	F	M
**Tukey Grouping**	A	B	A	B	A	B	A	A	A	A	B	A	B	A
**Fisher Grouping**	A	B	A	B	A	B	B	A	A B	A B	B	A	B	A
**Mean (°)**	-23.25	-27.72	13.36	4.60	16.61	10.7	10.21	17.19	15.73	10.89	-20.23	-13.65	5.36	9.11

**Table 5:** Header statistics results summary. ’C’ denotes central, ’L’ lateral, ’F’ female, ’M’ male, ’D’ dominant, and ’ND’ nondominant.

Header	Hip flexion		Hip abduction		Internal hip rotation				Knee adduction	
**ANOVA p-value**	0.006		0.031		<0.001		<0.001		0.002	
**Factor**	Gender		Leg		Leg		Gender		Gender	
	F	M	D	ND	D	ND	F	M	F	M
**Tukey Grouping**	A	B	A	B	B	A	A	B	B	A
**Fisher Grouping**	A	B	A	B	B	A	A	B	B	A
**Mean (°)**	54.37	42.26	-9.48	-12.94	0.82	7.40	7.45	0.77	-2.64	1.02

**Table 6:** Instep kick statistics results summary. ’C’ denotes central, ’L’ lateral, ’F’ female, ’M’ male, ’D’ dominant, and ’ND’ nondominant.

Instep kick	Hip flexion		Hip adduction		Dorsiflexion		Knee adduction	
**ANOVA p-value**	0.034		0.016		0.022		0.013	
**Factor**	Lateral-Central		Lateral-Central		Lateral-Central		Lateral-Central	
	L	C	L	C	L	C	L	C
**Tukey Grouping**	B	A	B	A	A	B	B	A
**Fisher Grouping**	B	A	B	A	A	B	B	A
**Mean (°)**	39.63	48.26	15.47	22.45	-16.46	-29.68	-1.385	4.167

### 3.1 Change of direction propulsive phase

In the propulsive phase for the change of direction, significant kinematic differences based on gender were reported for internal hip rotation (7.53° difference, p = 0.002), subtalar pronation (8.98° difference, p = 0.002), and knee abduction (3.69° difference, p < 0.001); the hip and ankle angles had larger magnitudes for female participants, while males showed greater range of motion in the knee. Leg side dominance also showed significant differences in knee abduction (1.71° difference, p = 0.017), where the nondominant leg showed a greater range of motion compared to the dominant leg. On-field positioning/gender interaction showed significant differences for hip abduction (p = 0.017).

### 3.2 Dribble propulsive phase

During the propulsive phase while dribbling, significant kinematic differences were observed based on gender in knee abduction (3.75° difference, p < 0.001), where males showed a greater range of motion. Gender also showed significant differences in internal hip rotation (8.76° difference, p < 0.001) and subtalar pronation (6.58° difference, p = 0.013), where females showed a greater range of motion. Leg dominance showed significant differences in hip abduction (4.47° difference, p = 0.032) and dorsiflexion (5.91° difference, p = 0.035), while on-field positioning/gender interaction showed significant differences in dorsiflexion (p = 0.033). The nondominant side had a greater range of motion for the hip abduction while the dominant side had a greater range of motion for dorsiflexion.

### 3.3 Header landing phase

During the landing phase in headers, significant kinematic differences were found based on gender, specifically in knee adduction (3.75° difference, p = 0.002), hip flexion (5.91° difference, p = 0.006), and internal hip rotation (6.68° difference, p < 0.001). In hip flexion and internal hip rotation, females showed a larger range of motion, while the results for knee adduction showed that males maintained in the negative sign convention (abduction). Leg side dominance also showed significant differences for internal hip rotation (6.58° difference, p < 0.001) and hip abduction (3.46° difference, p = 0.031). The non-dominant side had a range of motion compared to the dominant side.

### 3.4 Shot supportive foot planting phase

The supportive foot planting for the instep kick reported significant differences across on-field positioning for knee adduction (5.55° difference, p = 0.013), hip adduction (6.98° difference, p = 0.016), hip flexion (8.63° difference, p = 0.034), and dorsiflexion (13.22° difference, p = 0.022); all displayed greater joint angle values for central positioned players compared to lateral positioned players.

## 4. Discussion

Several studies have investigated potential relationships between lower body kinematics and injury risk in a wide range of sports, including soccer [17, 19–21]. Certainly, knee angles have a direct effect on the risk on ACL injury, but hip and ankle joints can also have an indirect influence; for the hip, higher peak values of internal rotation, adduction, and flexion increase the stress on the ACL; for the ankle, increased dorsi-flexion and foot pronation can affect the mechanics of the lower body and increase injury risk [19–21]. In addition, significant differences in these lower body joint angles have been established based on gender. With the purpose of delving deeper into lower body kinematics in soccer players and its relationship with ACL injuries, two factors have been introduced in addition to the gender variances: leg dominance and on-field general positioning. To the best of the authors’ knowledge, this is the first lower body kinematics study to include these specific factors through recreation of the most common soccer movements that lead into ACL injuries [16, 18]. In this study, lower limbs angles were monitored at the hip, knee, and ankle. The effect of on-field position, leg dominance, and gender varied by each one of the four recreated in-game motions (change of direction, dribbling, header, and instep kick).

### 4.1 Change of direction and dribble in propulsive phase

The change of direction and dribbling tasks were identical motions, differing only in the inclusion of the ball. Therefore, several factors resulted in similar effects when analyzing the propulsion phase. Firstly, in terms of gender differences, female participants displayed internal hip rotation angles 7.53° larger than males in the change of direction (p = 0.002), and 8.76° greater during dribbling (p < 0.001). These results agree with the findings by Pollard et.al when monitoring internal hip rotation; female college soccer players had a 7° greater peak angle when compared to their male counterparts during the propulsive phase of the change of direction [21]. In addition, female participants exhibited greater subtalar pronation peak angles compared to the male participants for both the change of direction and dribbling by about 9° (p = 0.002) and 7° (p = 0.013) respectively. This falls in-line with previous studies that have found female athletes to have subtalar pronation peaks 5° greater than males when performing cutting tasks [19]. In contrast, male participants demonstrated larger mean peak values of knee abduction than the female participants for both the change of direction (3.68° difference, p < 0.001) and the dribbling (3.75° difference, p < 0.001) tasks. Both male and female participants had similar knee range of motion in the frontal plane which may suggest that females still had greater knee adduction, although it was not deemed statistically significant.

Secondly, regarding leg dominance, a significant effect was observed in knee adduction (p = 0.017) during the propulsive phase of the change of direction task: the nondominant side had greater peak (9.02°) compared to the dominant side (7.31°). Leg dominance also played a significant role in the dribble task for hip abduction: the nondominant side (-27.72°) showed a greater peak when performing the dribble task with values of -23.25° (p = 0.032). DeLang et.al tested collegiate level male soccer players for lower body asymmetry between limbs and found no significant differences in joint mobility at the hip, knee, and ankle [47]. Importantly, DeLang et.al tested maximum joint mobility, while the present study measured kinematics while performing specific soccer in-game motions. This difference can explain the findings for knee adduction during change of direction and hip abduction while dribbling; soccer players tend to have a preferred side to pass and shot, and therefore, we speculate that muscle development, control, and flexibility can differ in between limbs during these in-game motions.

### 4.2 Header landing phase

Similar trends were seen during the landing phase of the header. First, for gender differences, female participants showed a peak hip flexion of 54.37° while the men were 42.26° (p = 0.006). These results are comparable to a study conducted by Butler et.al that compared the same landing from a header; women had peak hip flexion of 53.1° while men had a mean peak of 43.6° [48]. Also, female participants showed hip internal rotation peak values 7° greater than male participants (p < 0.001), similar to Mclean et.al findings where female participants had internal hip rotation angles about 5° greater than males [19]. Differences between genders were also observed in knee adduction, male participants showed a mean peak of 1.02° while female had -2.64°(p = 0.002). These results showed that participants never actually reached knee adduction during the landing phase of the header task, but instead remained in abduction; the same trend was observed in the Mclean et.al study which also measured a 4° difference between genders [19].

Secondly, for leg dominance, players’ nondominant side showed greater peak angles for hip abduction as well as hip internal rotation. Previous studies have found that the range of motion in the knee and hip joints were significantly lower in the non-dominant than in the dominant when landing [49]. This apparent contradiction could be due to the in-game nature of the motion recreated in the present study, especially in comparison to the single-leg landings performed in the study of Wang et al. The two-leg landing header recreated in the present study allowed for analysis of naturally occurring landing in contrast with controlled single landing. This allowed analysis of both legs during one motion, where the more developed leg facilitated the landing thus benefiting the nondominant side in the process. Wang et.al also mentioned that for the female soccer players, the risk of ACL injury can be higher in the non-dominant than in the dominant leg during unilateral dynamic movements [49]. This reasoning may be applied to bilateral motions as well, but likely not to the same extent since the dominant side is present and naturally makes up for the nondominant sides’ shortcomings through increased levels effort.

### 4.3 Shot supportive foot planting phase

Notably, the landing phase of the supporting foot during the instep kick resulted in significant differences between central and laterally positioned players in knee, hip, and ankle angles; each of which centrally positioned players demonstrated greater peak values. Specifically for knee adduction (5° difference, p = 0.013), both hip flexion (9° difference, p = 0.034) and adduction (7° difference, p = 0.016), as well as dorsiflexion peak mean values (12° difference, p = 0.022). Remarkably, laterally positioned players could not even reach knee abduction since they displayed mean peak values of -1.39° which is considered knee adduction. These findings suggest that central players tend to have a greater range of motion when performing tasks such as instep kicking. This could be due to the more dynamic nature of central players movements, especially center midfielders: their role on the pitch involves covering more ground than lateral players, such as a winger, in all directions resulting in quick accelerating/decelerating movements and rapid change of direction [32–35]. Lateral positioned players (winger, fullbacks, wide midfielders, wing back) appear to have fewer dynamic movements in game than central positioned players (strikers, center forward, midfielders, center backs, sweeper) as that they tend to stick by the sideline for the most part, sprint longer distances, and occasionally cut [36]. At a high playing level, this could potentially result in natural further development of specific muscles such as hip flexors, adductors, gluteus maximus, hamstrings, gastrocnemius, and soleus [50], reducing injury risk overall especially in non-contact scenarios. We consider this possibility since soccer teams tend to train collectively carrying out the same exercises, apart from goalkeepers who are separated for specific training. Therefore, the differences in muscle group development between players are not due to the uniform training across the roster, but from the specific on-field tasks during games.

Additional trends were also observed in the interaction of this on-field positioning factor with gender. Centrally positioned male participants in both tasks displayed greater joint angle peaks in comparison to their female counterpart while laterally positioned female participants displayed greater peak values than the lateral male participants. This trend was seen specifically in the hip abduction and dorsiflexion angles for the change of direction and dribbling tasks, respectively. Studies involving a cut without the ball yielded similar results; Mclean et al. found that the women demonstrated greater dorsiflexion peaks than men. Mclean et al. also found that men have slightly larger hip abduction than the female participants, in accordance to what was found in the present study [19]. The interaction with on-field positioning may have resulted in the greater difference seen in the present study of 7° (p = 0.017). Similarly, female players in lateral positions consistently had greater peak values than their centrally positioned counterparts in these tasks and joints. This is inconsistent with the findings regarding on-field positioning, where centrally positioned players consistently demonstrated greater peaks than the lateral players; this holds true for the male participants in the change of direction task both with and without the ball. This seemingly contradictory finding may be because the female and male participants were recruited from teams that, naturally, have different formations and styles of play. In this case, the women’s team seems to have more lateral players that are more comfortable in cutting maneuvers compared to the men’s team, which can be an indicative for inverted positioning based on their dominant foot (i.e., left winger on the right side). This certainly can be included as a limitation of the study since different team play styles can affect how often lateral players cut or if they’re expected to at all. Another limitation of this study includes the fact that the men and women tested play in different leagues pertaining to different federations which can result in varied competitive level. However, the inclusion criteria ensured that participants had a comparable training level since the sub-elite men’s team included players who had formed part of either NCAA division 1 or professional soccer in the past.

## 5. Conclusions

In summary, this study found significant kinematic differences between male and female soccer players as well as dominant and nondominant lower extremities. Differences have also been found across on-field positioning: knee adduction, hip flexion, hip adduction, and dorsiflexion differences during the instep kick between central and lateral positioned players, as well as other interactions with gender, were attributed to natural muscular development differences caused by variation of their typical in-game activity. Asymmetries in the lower limb may suggest increased risk of ACL injury during these maneuvers, and therefore, it could be reasonable to suggest implementing more position-specific ACL prevention programs since the more dynamic motions associated with central players seem to develop specific muscles differently compared to lateral players.

## Supporting information

S1 Raw data (XLSX)
